# Contributory Factors to Self‐Disclosure in Clinical Supervision: A Meta‐ethnography

**DOI:** 10.1002/cpp.70068

**Published:** 2025-03-26

**Authors:** Alina Elena Apostol, Kellie Turner, Rosa Hoshi, Aimee Pudduck

**Affiliations:** ^1^ School of Psychology Cardiff University Cardiff UK; ^2^ Aneurin Bevan University Health Board St Cadocs Hospital Newport UK

**Keywords:** clinical supervision, disclosure, meta‐ethnography, non‐disclosure, psychological therapist, psychologist

## Abstract

Research on supervisee disclosure in clinical supervision has predominantly focused on supervisees' tendency to withhold important information (e.g., negative feelings, perceived power differentials, clinical mistakes, personal issues and countertransference), highlighting a significant gap in understanding the factors that influence supervisees' self‐disclosure. Self‐disclosure, which is considered essential for supervisors to provide personalised feedback and tailored guidance, plays a critical role in effective supervision but remains underexplored in terms of its facilitators and barriers. This study addresses this gap by systematically exploring the contributory factors affecting supervisee self‐disclosure within the context of clinical supervision. Using the principles of meta‐ethnography, this systematic review synthesised findings from eight qualitative studies involving 180 participants (the sample ranging from 3 to 110). Through a thorough process of data extraction, translation, and synthesis, a conceptual framework was developed, positioning self‐disclosure as a dynamic process shaped by the interplay between supervisory dynamics, contextual factors, and supervisees' internal experiences. Key factors influencing self‐disclosure included the quality of the supervisory relationship, supervisees' perception of supervisors' personal characteristics, the emotional impact of self‐disclosure on supervisees and power differentials. These findings highlight the relational and systemic factors shaping supervisee self‐disclosure. Implications include strategies to improve supervisory relationships, reduce power imbalances and foster supportive environments. The study informs future research, enhances supervisory practice and guides training programmes to improve clinical supervision effectiveness.

**Summary**

- Supervisors' implicit behaviours and power dynamics shape disclosure: Perceived omniscience, inflexibility and reluctance to share personal experiences can reinforce supervisee non‐disclosure.
- Supervisees manage disclosure strategically: They withhold information to protect their image and avoid negative consequences. Rather than just fearing judgement, they assess supervisor receptivity and weigh risks, withholding information when openness feels unsafe or unproductive.
- Supervisory approach matters: A rigid, authoritarian style discourages disclosure, while a collaborative, empathetic approach fosters openness.
- Context influences disclosure: Factors like countertransference with clients and dual supervision (i.e., the integration of both managerial and clinical supervision) can impact supervisees' disclosure.
- Training must evolve: Supervision models should emphasise the need for reciprocal vulnerability and responsiveness to supervisees' disclosure needs.

## 1 Introduction

Research literature on clinical supervision and its effectiveness on supporting the safe practice and professional development of psychological practitioners has grown considerably (APA 2015; Chircop Coleiro et al. 2023; Falender et al. 2014). An important factor of clinical supervision is how much information supervisees share with their supervisors (i.e., supervisee disclosure). In this literature review, the terms ‘supervisees’ and ‘supervisors’ refer to trainees or qualified psychological practitioners, such as clinical/counselling psychologists and/or psychological therapists. Supervisee disclosure refers to the sharing of information about the client, therapeutic and supervisory interactions, and personal experiences (Ladany et al. 1996). Effective supervision relies on multiple sources of information to assess supervisee performance, including direct observation through video recordings and supervisee disclosure (Bernard and Goodyear 2014; Callahan et al. 2009; Falender and Shafranske 2012; Watkins 2020). While video recordings provide direct insight into clinical work, they are not always feasible or routinely used in supervision (Bernard and Goodyear 2014). Consequently, supervisee disclosure remains a primary means for supervisors to provide individualised feedback and ensure competent practice (Knox 2015; Watkins 2020).

Research suggests that supervisees commonly withhold details about clinical mistakes, negative perceptions of clients and concerns about supervision itself (Cook et al. 2020; Hess et al. 2008; Mehr et al. 2015). For instance, Ladany et al. (1996) found that 97.2% of therapists undergoing clinical and counselling psychology training refrained from disclosing negative feelings towards their supervisors, with 53% of these being discussed with peers or friends in the field instead. About 44% chose to withhold information regarding clinical mistakes, such as forgetting to follow up on referrals or not completing extensive risk assessments when they should have. Participants admitted to withholding information in about 8.06 instances during an average of 15 supervision sessions. Mehr et al. (2010) further reported that 84.3% supervisees engaged in non‐disclosure, often due to concerns about negative evaluation or perceived inappropriateness and irrelevance of topic. Impression management, or the desire to maintain a favourable professional image, was a key driver of non‐disclosure, also reported in later studies (Cook et al. 2018; Goffman 1956, 2023).

Existing qualitative systematic reviews have explored supervisee disclosure and non‐disclosure in clinical supervision (Chircop Coleiro et al. 2023; Falender et al. 2014). Falender et al. (2014) examined supervision best practices but did not fully address the complexities of supervisee non‐disclosure or the factors that influence it. More recently, Chircop Coleiro et al. (2023) provided a systematic synthesis of qualitative research on supervisee disclosure; however, their review primarily focused on the content of disclosure rather than the underlying psychological and relational mechanisms influencing non‐disclosure. Additionally, their synthesis did not thoroughly examine the implications of non‐disclosure for supervisory relationships or client care. These limitations highlight the need for a more integrative approach to understanding supervisee non‐disclosure, particularly its impact not only on supervisory relationships but also on how non‐disclosure may shape supervision dynamics, professional development and clinical outcomes.

Supervisee non‐disclosure is influenced by various factors, including supervisor characteristics (e.g., inflexibility), relational behaviours (e.g., not exploring supervisee's feelings), power differentials and the quality of the supervisory relationship (Cook et al. 2020; Hutman and Ellis 2020; Meydan 2020; Singh‐Pillay and Cartwright 2018; Taylor and Ellis 2023). Studies highlight that supervisor rigidity and a lack of relational attunement contribute to non‐disclosure (Meydan 2020; Zamir et al. 2022; Žvelc and Žvelc 2020). Supervisees' non‐disclosure results from concerns around shame and risk of criticism from their supervisors as well as not feeling safe enough in the supervisory relationship (Žvelc and Žvelc 2020). Non‐disclosure in supervision can lead to harmful clinical practice, contributing to potential blockages and/or unresolved therapeutic and supervisory ruptures (Knox 2015; Ladany et al. 2013). Consequently, non‐disclosure can undermine supervision quality, leading to ineffective clinical practice and unresolved supervisory ruptures (Knox 2015; Ladany et al. 2013).

Beyond clinical work, self‐disclosure extends to supervisees' lived experiences of mental health difficulties, which can significantly impact their professional development and therapeutic practice (Barnett et al. 2007; Boyle and Kenny 2020; Bradley and Becker 2021; Falender and Shafranske 2012; Gelso and Hayes 2007; Goldberg et al. 2015; Gray et al. 2001; Hess et al. 2008; Hess‐Holden 2019; Staples‐Bradley et al. 2019; Walsh et al. 2002). Integrating lived experience in clinical supervision may prevent potential consequences such as overidentification, vicarious trauma, triggering of supervisee's mental health symptoms and compassion fatigue (Cleary and Armour 2022; Rothschild 2006; St. Claire and Clucas 2012). However, supervisees hesitate to disclose personal mental health challenges due to stigma and fear of discrimination (Cleary and Armour 2022; Devendorf 2022; Salzer 2022; K. Turner et al. 2021). While mental health disclosures may enhance empathy and supervisory relationships, they require careful navigation to ensure they benefit both the supervisee and the supervisory process (Cleary and Armour 2022; Hill et al. 2018).

Mental health practitioners have conflicting thoughts regarding disclosures of lived experience of mental health difficulties (Hinshaw 2008; Kimhy et al. 2022; Prinstein 2022), with 36% being ‘cautious about disclosure’ to colleagues including supervisors (Boyd et al. 2016). While some researchers openly discuss their experience of working in mental health services with a diagnosis of a mental health difficulty (Deegan and Affa 1995; Deegan et al. 2017; Frese et al. 2009; Kemp et al. 2020), the prevailing culture of ‘don't ask, don't tell’ still persists (Byrne et al. 2022). The reluctance to disclose is mostly linked to shame, perceived lack of competency and career concerns (Cleary and Armour 2022; Gras et al. 2015; Harris et al. 2016; Tay et al. 2018; K. Turner et al. 2021). Non‐disclosures of lived experience could perpetuate further discrimination and stigma, hindering help‐seeking behaviours in supervision (Byrne et al. 2022; Harris et al. 2016; Mental Health Foundation 2021).

Some psychologists view clinicians' lived experience positively (Cleary and Armour 2022; Devendorf 2022; Kemp et al. 2020; Kimhy et al. 2022; Victor et al. 2022). Cleary and Armour (2022) highlight that sharing lived experience in clinical supervision offers numerous benefits for supervisees, such as enhancing empathy and understanding clients better, as they can relate to the challenges faced by individuals with mental health difficulties. Such disclosures also nurture greater supervisory and therapeutic alliances, as it may facilitate a more trusting relationship (Cleary and Armour 2022; Hill et al. 2018). Harris et al. (2016) state that supervisees' openness to disclose their own lived experience of mental health may challenge the culture of non‐disclosure and encourage a more open approach to it, reducing stigma. Cultivating authenticity and supportive environments within supervisees' supervisory experiences may positively influence work colleagues to navigate their own professional and personal challenges (Knox et al. 2011; Ladany et al. 2013). This could promote further professional growth, as disclosures may create more space for reflection around therapist biases, increasing self‐awareness and understanding of the potential impact of lived experience on therapeutic relationships (Knox et al. 2001).

Prominent supervision models emphasise the role of disclosure in professional growth. The discrimination model (Bernard 1979) outlines how supervisors function as teachers, therapists and consultants to foster supervisee openness. In the role of a teacher, supervisors provide direct instruction and guidance. As therapists, they attend to the emotional and personal aspects of the supervisee's experiences. In the consultant role, supervisors collaborate with supervisees to explore issues and develop solutions. The integrated developmental model (Stoltenberg and McNeill 2010) complements this approach by emphasising the need to tailor supervision to the supervisee's developmental stage (Stoltenberg and McNeill 2010). Early‐stage supervisees, who are typically anxious and dependent, require a highly supportive and non‐judgemental environment to feel safe enough to disclose their experiences and mistakes. As supervisees gain confidence and move to intermediate stages, supervisors should provide a balanced mix of support and challenge, encouraging supervisees to reflect more deeply on their clinical work and personal experiences. For advanced‐stage supervisees, who are more autonomous and confident, supervisors should promote deeper self‐reflection and self‐disclosure, facilitating ongoing professional growth and self‐awareness. Both models highlight that while supervisee disclosure is not the sole means of obtaining supervisory feedback, it remains integral to supervision effectiveness, complementing other evaluative methods like video recordings (Bernard 1979; Stoltenberg and McNeill 2010).

### 1.1 Rationale

Research on supervisee disclosure in clinical supervision has primarily focused on the supervisees' tendency to withhold important information, such as negative feelings and clinical mistakes (Chircop Coleiro et al. 2023; Cook et al. 2019; Falender et al. 2014; Knox 2015; Ladany et al. 1996). However, the implications of disclosure remain underexplored, particularly regarding its impact on the supervisory relationship and clinical practice, potentially leading to clinical errors and hindered professional development (Knox 2015; Ladany et al. 2013; Mehr et al. 2010; Spence et al. 2014; Zamir et al. 2022). Synthesising and interpreting qualitative studies within this topic is crucial for several reasons: It can identify specific areas requiring further research, inform supervisor training programmes and enhance the overall quality of supervision. Additionally, by addressing the factors contributing to non‐disclosure, future research can help to establish more effective supervisory practices that foster openness and trust. This systematic review aims to provide an integrative perspective on supervisee disclosure, addressing the limitations of prior reviews and enhancing the understanding of this phenomenon. This study specifically focuses on synthesising qualitative research related to the nuances of disclosure in clinical supervision, contributing to a deeper understanding of the phenomenon and its implications for clinical practice.

### 1.2 Aims

This systematic review aims to answer the following research question: *What are the factors that facilitate or prevent supervisees' self‐disclosure in clinical supervision?*

## 2 Methodology

This systematic review study comprised of conducting a systematic literature search, critically appraising studies included and synthesising the gathered data using meta‐ethnography (Noblit and Hare 1988). The protocol for this study was registered on the International Prospective Register of Systematic Reviews (i.e., PROSPERO) on 14 June 2023 with registration number CRD42023395113. No protocol amendments were made postregistration.

### 2.1 Systematic Literature Search

An initial database search was conducted in May 2023, followed by another comprehensive search in October 2023 to ensure no articles were missed. Literature scoping was also done prior to the systematic search to find articles that were potentially relevant. The systematic literature search aimed to identify qualitative studies investigating disclosure and non‐disclosure in clinical supervision. The research question was defined using the PICO (Problem, Intervention, Comparison and Outcome) framework (Table 1; Aslam and Emmanuel 2010; Sackett 1997). The Preferred Reporting Items for Systematic Reviews and Meta‐Analyses (PRISMA) checklist guideline was used to ensure a transparent and comprehensive reporting of systematic review (Liberati et al. 2009; Page et al. 2021), including information regarding the identification, selection and critical appraisal of studies identified. The search terms were developed through discussions with the research team and the university Subject Librarian as well as initial searches of the author's scoping of literature. These agreed‐upon search terms were subsequently combined within each concept using the Boolean operator. Database searches were undertaken on PsycINFO (via Ovid), MEDLINE (via Ovid), Embase (via Ovid) and Web of Science, using the same search strategy across each database. Grey literature was not considered as it can pose challenges in the synthesis process due to limited quality control, accessibility issues, heterogeneity in reporting and publication bias (Adams et al. 2016; Benzies et al. 2006; Franks et al. 2012; Godin et al. 2015; Mahood et al. 2014; A. Turner et al. 2005).

**TABLE 1:** The PICO framework.

P (problem/population)	I (intervention/exposure)	C (comparison)	O (outcome)
Supervisee (e.g., psychologists, psychological therapists and trainees)	Clinical supervision	Disclosure and non‐disclosure factors	Safe and contained disclosure in clinical supervision

**FIGURE 1:**
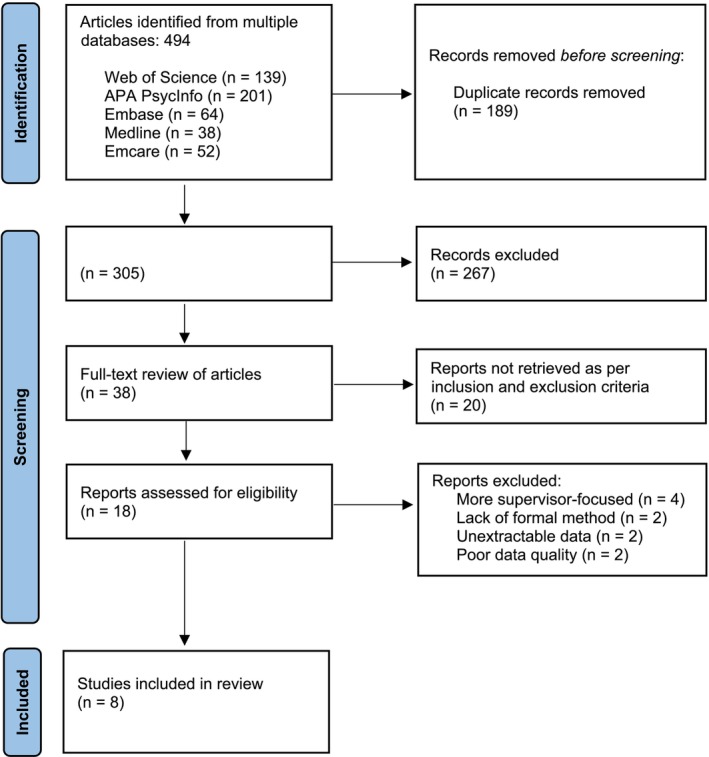
PRISMA flow diagram of the systematic search strategy.

Papers retrieved from the main search (*N* = 494) were screened by Rayyan, and duplicate entries were eliminated (Ouzzani et al. 2016). To ensure reliability and validity of the reviewing process, all titles and abstracts of remaining papers (*N* = 305) were screened by two independent reviewers in accordance with the inclusion and exclusion criteria agreed in supervision (Table 2). This helped to reduce the likelihood of errors and individual biases and enhanced consistency of the screening process (Liberati et al. 2009). After consulting with the research team, no restrictions were imposed on the publication year of the studies included in the screening process, with years of publication varying from 1994 to 2023. The inter‐rater reliability was initially at 96.1%; however, after differences were discussed and resolved, 100% agreement was reached. The included studies were not restricted to articles published in English language only unless full intelligible translations were unavailable. A full‐text review was carried out by the primary reviewer on articles meeting the inclusion criteria through title and abstract screening. A second independent reviewer assessed 15% (*N* = 6) of the full‐text articles for inclusion. Initially, there was 83% inter‐rater agreement (five out of six papers), but after additional clarification of exclusion criteria, complete agreement was achieved. The outcomes of the search process at each stage were documented and reported with the PRISMA flowchart (Figure 1).

**TABLE 2:** Inclusion and exclusion criteria.

Inclusion	Exclusion
Qualitative studies of various research methods including interviews, focus groups, case studies and open‐ended questionnaires	Exclusively quantitative studies
Mixed‐method studies where the qualitative data are extractable	Mixed‐method papers where the qualitative data are insufficient or not extractable
Research focusing on supervisees' experience of self‐disclosure in clinical supervision	Research that do not address the experience of self‐disclosure in clinical supervision
Studies clearly include perspectives of supervisees within the psychology and/or psychological therapy professions including trainees/interns.	Studies exclusively investigating perspectives of supervisees outside of the targeted population (e.g., nurses and medics) or supervisors
Primarily empirical research, peer‐reviewed articles	Studies which are not primarily empirical research (e.g., systematic reviews, books and policy reports)
Research with full text available in the English language	Research with partial or full text available in any language other than English

### 2.2 Critical Appraisal of Studies

The systematic review process included the use of a quality appraisal tool, the Critical Skills Appraisal Programme (CASP) checklist (Noyes et al. 2008). CASP assesses key principles and evaluation criteria of qualitative research, including a clear statement of purpose, suitability of methodology, study design and recruitment strategy, procedure employed for data collection, acknowledgment of the researcher–participant relationship, ethical considerations, data analysis rigour, clarity of findings, and the overall contribution and significance of the research. CASP checklist lacks a designated scoring system; therefore, a basic scoring system used in previous systematic reviews was used (Charles et al. 2020; Ibrahim et al. 2020; Matthews et al. 2018; D. Patton 2022) as well as the reporting of the actual domains and study characteristics. A score of one point was assigned for each ‘yes’, and zero points for each ‘no’. Questions rated as ‘cannot tell’ or ‘partially met’ were allocated half a point, consistent with recommendations from Toye et al. (2014) for meta‐ethnography research. With Question 10 excluded from formal rating due to its highly subjective nature, each study had a maximum possible score of nine (Table 3). Studies were also graded from A to C based on their methodological quality, as commonly done in other metasyntheses (Graham et al. 2020; D. Patton 2022). A proportion of articles included in this study (*N* = 3, > 25%) were inter‐rated by a second reviewer using the CASP tool, as part of the integral systematic review process. The inter‐rater agreement was 100%. The reviewer was a trainee clinical psychologist with no direct involvement in this project.

**TABLE 3:** CASP scoring system.

Grade	Likelihood of methodological flaws	Score on CASP
A	Low	8.5 or above
B	Moderate	5 to 8
C	High	Less than 5

The decision to include or exclude papers in this review was not solely determined by the quality appraisal rating. Atkins et al. (2008) highlighted that quality appraisal ratings often reflect the quality of the written report rather than the study itself and that the richness of data is more important during the quality appraisal, for example, the use of semi‐structured interviews and thematic analysis, rather than descriptive studies that lack in‐depth qualitative data and provide few quotes related to participants' experiences (Knowles et al. 2014). For example, two mixed‐method papers considered for this systematic review appeared to have extractable qualitative information. However, the data reported lacked sufficient detail (Table 6), richness of insights and theoretical contribution, failing to meet the standard qualitative research benchmark (Kidder and Fine 1987, as cited in Harper and Thompson 2012).

### 2.3 Data Synthesis

The qualitative analysis used for this review was the Noblit and Hare (1988) metaethnography method. This seven‐phase methodology aims to provide a deeper understanding of a phenomenon by systematically reviewing and interpreting findings from multiple qualitative studies. This refined approach involves the translation of concepts across different published findings, leading to the development of new insights and theories (Britten et al. 2002). Metaethnography is widely recognised as a valuable method for synthesising qualitative studies within healthcare research (Campbell et al. 2003; Ring et al. 2011). Given the nature of the chosen studies for this paper (e.g., qualitative data), meta‐ethnography was considered the most appropriate and comprehensive method for qualitative synthesis. The author of this study followed mainly the guidance of Noblit and Hare (1988) seven‐phase approach to meta‐ethnography (Table 4). Throughout the process of synthesis, guidance was drawn from the worked examples of meta‐ethnography (Atkins et al. 2008; Britten et al. 2002; D. Patton 2022). In stages three to five of the process, detailed tables were developed for each paper to facilitate the collation, review and comparison of the studies. While similarities across cases are essential for reciprocal translation, there should also be an openness to explore differences or exceptions (Noblit and Hare 1988). Thus, the Noblit and Hare (1988) principle of ‘one case is like enough, except that …’ was considered. In other words, the reviewer took into account the cases presented in tables and original texts, acknowledging that certain aspects might reveal variations or nuances among them.

**TABLE 4:** Author's seven‐phase approach to metaethnography as per Noblit and Hare (1988).

Phase	Description	Current study methods
1	Getting started	Identifying areas of interest, involving consultation with supervisors and checking existing reviews to avoid duplication
2	Deciding what is relevant	Defining clear inclusion and exclusion criteria after identifying a specific area of interest. Developing a search strategy with Boolean operators, guided by consultation with a Subject Librarian. Registering the review with PROSPERO
3	Reading the studies	Reading studies repeatedly to familiarise oneself with key concepts. Conducting quality ratings and discussing ratings with a secondary rater. Organising data into first‐ and second‐order constructs
4	Determining how studies are related	Identifying and describing metaphors/concepts within the studies. Presenting second‐order concepts from included studies in a table for further comparison (see Table 4). Using ‘concept maps’ to support the development of relationships
5	Translating studies into one another	Constantly comparing identified concepts. Creating a grid for clear comparison of concepts endorsed across studies. Identifying similarities and differences; employing reciprocal translations as no refutational translations were identified. Organising concepts into abstracted conceptual categories/framework
6	Synthesising translations	Developing a line of argument by integrating translations into a conceptual model. Creating a visual structure of developed conceptual categories
7	Expressing the synthesis	Expressing the synthesis in written form, complemented by a visual representation of conceptual categories

Considering Schutz (1962) notions of first‐, second‐ and third‐order constructs, the second‐order constructs were the primary ‘data’ and ‘building blocks’ of this meta‐ethnography (Britten et al. 2002, as cited in D. Patton 2022). These constructs were then abstracted further to create third‐order constructs, representing the reviewers' interpretations of the original authors' interpretations. While first‐order constructs often depict participants' interpretations in their own words, Toye et al. (2014) caution against using them in meta‐ethnographies. The individual quotations chosen by researchers likely encapsulate larger datasets, becoming second‐order interpretations. Also, introducing first‐order constructs in meta‐ethnographies poses risks of potential reinterpretations and incorrect attributions of new meanings by the current reviewer. Consequently, to ‘preserve meaning from original texts as far as possible within qualitative synthesis’ (Walsh and Downe 2006, as cited in D. Patton 2022), this review focused on second‐order constructs. This approach enabled a nuanced and contextually sensitive analysis, encompassing both shared characteristics through reciprocal translation (i.e., matching concepts with others) and unique distinctions through refutational translation (i.e., reviewing instances of data opposition). When no instances of disagreement were identified, reciprocal translations were employed across the entire data set. The reviewer developed a line of argument through a process of reinterpretation of third‐order constructs (existing interpretations) and a thorough comparison of these interpretations. Direct quotes representing participants' self‐interpretations of experiences (first‐order constructs), researchers' interpretations of participants' understandings of experiences (second‐order constructs) and the present reviewer's interpretation of both first‐ and second‐order constructs (third‐order constructs or synthesised themes) were applied to create a new theoretical understanding of the data (Schutz 1962). This process concluded with the integration of findings within a new theoretical model.

### 2.4 Ethical Considerations

This meta‐ethnography study did not involve direct engagement with human participants. Ethical guidelines for secondary data analysis were followed, ensuring accurate representation, proper attribution and respect for original findings. No personal data were used, and no risks were posed, so formal ethical approval was not required.

## 3 Results

The study selection process followed the PRISMA model (Figure 1). A total of 494 articles were identified across multiple databases (Figure 1). After removing duplicates, 305 articles underwent abstract review. From these, 38 articles were examined in full text, applying the inclusion and exclusion criteria (Table 2) and critical appraisal methods. This resulted in the inclusion of eight studies.

### 3.1 Study Characteristics

This systematic review comprises eight studies involving a total of 180 supervisees, with samples ranging from 3 to 110 participants. Study characteristics were summarised (Table 5). The studies varied in the depth of demographic information provided, including age, ethnicity, years of training and duration of supervisory relationship. Gender distribution was reported in all studies, with 140 female, 35 male, 3 non‐binary and 2 undisclosed genders. Except Spence et al. (2014), all studies provided participant ages, ranging from 21 to 60 years old. However, two studies presented average ages instead of age ranges: 25 and 43 years (Cook et al. 2019 and Singh‐Pillay and Cartwright 2018, respectively). Most studies focused on students/trainees in master/doctoral programmes related to counselling or clinical psychology. Two studies included newly qualified psychologists. The majority of studies were conducted in Western cultures, except for two conducted in Turkey and South Africa. Race and ethnicity data were included in all but two studies (Meydan 2020 and Sweeney and Creaner 2014 respectively). The studies examined disclosure in clinical supervision, covering various aspects such as the supervisory relationship, clinical issues, negative reactions and power dynamics. Most studies focused solely on qualitative findings, except for two: Hess et al. (2008) included numerical data to distinguish ‘good’ versus ‘problematic’ supervisory relationships; Cook et al. (2018) used Chi‐square to explore response bias by comparing participants who answered open‐ended questions with those who did not. While these two studies used mixed methods, qualitative analyses could be extracted from the results, meeting the benchmark of qualitative research (Kidder and Fine 1987, as cited in Harper and Thompson 2012). Data collection methods varied among the studies, with semistructured interviews being the most common. Two studies utilised the interpersonal process recall (IPR) approach (Kagan 1980; Larsen et al. 2008) for interview schedules, while others relied mainly on open‐ended questions. Data analysis methods differed, with six approaches used across the eight studies. The rationale and appropriateness of methodologies varied, influencing some studies' lower CASP ratings.

**TABLE 5:** Characteristics of studies included, key findings and CASP ratings.

Number of study	Authors, year and study country of origin	Title	Aims of study	Sampling method	Sample characteristics (e.g., size, gender, age, ethnicity, supervisee role and supervisory relationship duration)	Data collection method	Data analysis	Key findings	CASP quality rating
1	Hess et al. (2008) USA	Predoctoral interns' nondisclosure in supervision	‘To explore predoctoral interns experience of non‐disclosure’ ‘To explore reasons for intentional non‐disclosure’ ‘To investigate the content of intentional non‐disclosure’ ‘Questioned whether there were factors that would have facilitated supervisee disclosure’ ‘Examining what effects, if any, interns thought their non‐disclosure had on their personal development as well as on their supervisory relationship’ ‘Understanding the context of supervisees' non‐disclosures’	Purposive sampling—through personal contacts with interns and training directors at university counselling centres from nine different East Coast states in the United States. All training programmes were approved by the American Psychological Association (APA).	*N* = 14 predoctoral interns (11 female, 3 male; 10 European American/White [non‐Latino], 2 African American, 2 Asian American)—13 in a counselling psychology PhD and one in a clinical psychology PsyD Age range—27 to 38 years (*M* = 31.21, SD = 3.68) Supervisory relationship duration was not reported.	Semistructured interviews on the basis of Shirley A. Hess review of the literature and her personal supervision experiences	Consensual qualitative research (CQR; Hill et al. 1997; Hill et al. 2005)	*The context*: ongoing problematic supervisory relationship or positive supervision experience *Supervisory relationship*: safe or unsafe relationship; comfortable/uncomfortable disclosing; intern valuing supervisor's supervision style and supervisor's expertise *Content of non‐disclosure*: clinical issues; problems in supervisory relationship *Reasons for non‐disclosure*: concerns about evaluation; afraid of hurting supervisor's feelings; power differentials *What would have helped intern disclose*: supervisor asking/disclosing incident *Perceived effects of non‐disclosure*: negative or positive factors	9 (A)
2	Sweeney and Creaner (2014) Ireland	What's not being said? Recollections of nondisclosure in clinical supervision while in training	‘What factors lead to non‐disclosure in a supervisory relationship?’	Purposive sampling—through emailing counselling psychology graduates, 2‐year post training from a counselling psychology training programme which was accredited by the Psychological Society of Ireland (PSI). All participants were members of PSI and shared a common training experience.	*N* = 6 counselling psychology graduates, 2‐year post training (three female, three male) Age range—28 to 55 years (participant mean and standard deviation values for age were not reported) Supervisory relationship duration was not reported.	Semistructured interviews on the basis of feedback from a pilot interview upon which questions were modified	Consensual qualitative research (CQR; Hill et al. 1997; Hill et al. 2005)	The nature of difficulty: context (i.e., satisfied or dissatisfied); content (e.g., clinical issues and transference); supervision concerns *Reasons for non‐disclosures*: supervisor's nonfacilitative behaviour; previous attempt to disclose unsuccessful; stage of training and developmental level; evaluation; power dynamic; theoretical orientation; organisational pressure *The supervisory relationship*: supervisee perception of helpful aspects; valued supervisor's style; perception of hindering aspects; lacked investment/insight/experience; unprocessed supervisory transference *Facilitative factors*: supervisor's actions and supervisee's actions; reduced own egotism; external factors	9 (A)
3	Spence et al. (2014) UK	Supervisee self‐disclosure: A clinical psychology perspective	‘To investigate qualified UK clinical psychology supervisees' use of voluntary self‐disclosure in supervision throughout their careers’ ‘To develop a theoretical understanding of supervisees' self‐disclosure processes’	Theoretical sampling—eight participants were recruited through a newly qualified continuing professional development (CPD) scheme from different services across four NHS trusts. Following emerging theory, two additional participants were recruited via the scheme's facilitator of a CPD meeting.	*N* = 10 newly qualified UK clinical psychologists (eight female, two male) Age range values were not reported. Duration of the current supervisory relationship was an average of 1.65 years since participants qualified as clinical psychologists.	Semistructured interviews on the basis of Spence review of the literature and personal supervision experiences. Supervision frequency varied, with the majority of participants receiving at least 1.5 h of supervision monthly.	Constructivist grounded theory (Charmaz 2014)	*Setting the scene*: supervision context; personal values; professional culture; understanding self‐disclosure *Supervisory relationship*: compatibility in the supervisory dyads' and theoretical orientation; ‘good enough’ compatibility *Using self‐disclosure*: assessing; non‐disclosure; self‐monitoring; take elsewhere; deciding how to self‐disclose *Reviewing the outcome of self‐disclosure*: building trust; consequences	9 (A)
4	Cook et al. (2018) USA	Counselor‐in‐training intentional nondisclosure in onsite supervision: A content analysis	‘What are the types of information that counsellors‐in‐training (CITs) intentionally withhold from their supervisors during their internship's onsite supervision?’ ‘What are the reasons for their non‐disclosure?’	Purposive sampling—through the assistance of counsellor educators of 14 CACREP‐accredited institutions. All training programmes were approved by the Council for Accreditation of Counseling & Related Educational Programs (CACREP). Participants included were currently enrolled in practicum internships as part of their training.	*N* = 110 CITs (88 female, 17 male, 3 non‐binary, 2 did not want to disclose their gender; 71 White [non‐Hispanic], 23 African American, 4 Asian/Pacific Islander, 3 Hispanic/Latinx, 3 multiracial, 1 Native American, 1 ‘none of the above categories’, 4 preferred to not disclose)—64 were enrolled in a clinical mental health counselling track, 32 in a school counselling track, 9 in a college counselling and school affairs, and 5 in a marriage, couples and family track. Age range—22 to 60 years (*M* = 28.13, SD = 7.43) Supervisory relationship duration was not reported.	Semistructured interviews consisting of two open‐ended questions and a 15 items questionnaire to collect demographic information about the participants and their current onsite internship supervisors	Content analysis (Hsieh and Shannon 2005)	*Type of intentional non‐disclosure*: negative reactions to supervisor; general client observations; clinical mistakes; client–counsellor attraction issues; countertransference; supervision setting concerns; personal issues; developmental needs; negative reactions to client; experiencing sexual harassment; a peer issues *Reasons for non‐disclosure*: impression management; negative feelings; supervisor not competent; perceived unimportant; deference; poor alliance with supervisor; supervisor agenda; did not want to harm client or confidentiality; too personal; pointlessness; consulted with another supervisor	9 (A)
5	Singh‐Pillay and Cartwright (2018) South Africa	The unsaid: In‐depth accounts of non‐disclosures in supervision from the trainees' perspective	‘To explore trainees' subjective experiences and perceptions of non‐disclosure in clinical supervision’	Purposive sampling—through a preliminary discussion. All participants were enrolled in a 1‐year supervised internship at either an university counselling centre or a hospital, as part of a Health Professional Council of South Africa (HPCSA) accredited training/master programme Counselling or Clinical Psychology	*N* = 8 intern psychologists/trainees (five women, three men; four White and four African) Age average—43 years The duration of their current supervisory relationship was an average of 6.25 months.	Semistructured interviews; however, it is unclear what informed the interview schedule	Interpretative Phenomenological Analysis (IPA; Smith et al. 2009)	*Purposeful non‐disclosure*: need to take control of power relations and imbalances in supervision *Perceptions and experiences that prevent and facilitate trainee disclosures*: power imbalances; perception of power; disempowerment *On learning from supervisor*: supervisor's modelled non‐disclosure; supervisor strategic self‐presentation *Implications for trainee learning and therapeutic practices*: non‐disclosure anxiety; therapeutic mistakes; apparent belief of ‘knowing better’; shame about underlying anxiety; ethical transgression	7 (B)
6	Cook et al. (2019) USA	Exploring supervisees' in‐session experiences of utilizing intentional nondisclosure	‘What are supervisees' in‐session experiences in the specific moments when they intentionally withhold information from their supervisors?’	Purposive sampling—through two authors inviting master's‐level practicum and internship students from two CACREP‐accredited counselling programmes in the southeast United States to participate in the study during their practicum and internship course	*N* = 10 master's‐level practicum and internship students (10 female; 8 White/Caucasian, 1 African American, 1 Latina/Hispanic)—six were enrolled in a school counselling track and four in a clinical mental health counselling track. Age range—27 to 38 years (*M* = 31.21, SD = 3.68) Supervisory relationship duration—not reported	Semistructured interviews informed by the interpersonal process recall (IPR; Kagan 1980) sample prompts included in Bernard and Goodyear (2014).	Transcendental phenomenological analysis (TPA; Moustakas 1994)	*Impressions of the supervisory relationship*: personal reactions to client work; not trusting supervisor; receiving critical feedback; feeling pressured to respond to supervisor's questions *Reactions to the structure or process of supervision*: pragmatic structural issues; individual supervision structure and style issues *Experiences of balancing professional and evaluative relationship*: desire to maintain professional relationship; concerns about evaluation	9 (A)
7	Foskett and Van Vliet (2020) Canada	Understanding supervisee nondisclosures in supervision with videorecording review and interpersonal process recall	‘What are trainees' experiences and internal processes of non‐disclosures in supervision sessions using videorecording review?’	Purposive sampling—through electronic mailing lists, brief on‐campus presentations and posters to first year internship students of a master's‐level counselling practicum in Western Canada	*N* = 3 master's‐level practicum and internship students (one female and two male; one Caucasian/European, one Latino and one Asian)—six were enrolled in a school counselling track and four in a clinical mental health counselling track. Age range—25 to 36 years (*M* = 30.67, SD not reported) Supervisory relationship duration was not reported.	In‐the‐moment review of a videorecorded supervision session using interpersonal process recall (IPR; Kagan 1980; Larsen et al. 2008) interview methods	Qualitative case study design (Merriam 2002).	*Validation*: supervisor's paraphrasing and reflecting back; feeling affirmed; encouragement and empowered *Safety*: trust and openness; nondefensiveness and vulnerability; sense of safety *Growth and accomplishment*: feeling confident in own practice; pleased for supervisor's observation of accomplishments *Performance anxiety*: heightened awareness of the evaluative nature of supervision; minimising or dismissing positive feedback; self‐judgement and self‐consciousness *Avoidance*: avoided confrontation or self‐assertion; wanting to respect the supervisor's expertise; becoming inpatient	9 (A)
8	Meydan (2020) Turkey	Turkish first‐time supervisees' disclosure and nondisclosure in clinical supervision	‘What are first‐time supervisees' opinions regarding disclosure and non‐disclosure in clinical supervision?’	Variation sampling—through an announcement to six supervision classes via supervisors of a guidance and counselling undergraduate programme within a Turkey‐based university	*N* = 19 first‐time supervisees within an individual counselling practice course (14 female; 5 male)—six were enrolled in a school counselling track and four in a clinical mental health counselling track. Age range—27 to 38 years (*M* = 31.21, SD = 3.68) Participants' counselling and supervision experiences ranged from 7 to 11 supervision sessions. Supervisees had no prior supervision experiences.	Semistructured interviews on the basis of the author review of the literature. Draft interview questions were informed by M. Patton (2002) guidance and reviewed by two researchers, both holding doctoral degrees in counselling psychology.	Content analysis (Schreier 2014)	*Disclosure*: supervisee needs; thoughts about supervisor *Non‐disclosure*: personal issues; supervision‐related issues; negative feelings about client *Reasons for disclosure*: supervisor's personal characteristics; supervisor's intervention (e.g., open‐ending questioning and encouragement) *Reasons for non‐disclosure*: supervisor's change/criticisms, authoritative and having negative attitudes towards supervisees *Outcomes*: effects on supervisee (self‐awareness, feeling relaxed and understood); effects on supervision (satisfaction; qualified environment)	7 (B)

### 3.2 Quality Appraisal Findings

The studies examined in this metaethnographic review were generally rated as having moderate likelihood of methodological flaws, scoring either 7 or 9 on the CASP tool. Key reasons for lower scores included insufficient details about the research design chosen and data analysis process, lack of rationale for the used methodology, limited information on ethical considerations and an absent account of researcher reflexivity. A prominent strength across all studies was the clear statement of research aims, appropriate recruitment and data analysis procedures, and comprehensive summaries of findings (Table 6). Seven out of eight studies used qualitative methodology, which was deemed appropriate based on the research aims. The papers with lower scoring, Meydan (2020) and Singh‐Pillay and Cartwright (2018), had similar limitations. Meydan (2020), which scored 7 on the CASP tool, had limited information on ethical considerations and data analysis process. The authors did not consider the relationship between researcher and participants, demonstrating a lack of reflexivity. Singh‐Pillay and Cartwright (2018) scored 7 on the CASP tool due to limited rationale around the chosen methodology and research design. The study also made very limited comments on ethical issues. Unlike Meydan (2020), in Singh‐Pillay and Cartwright (2018), researcher bias was explored and discussed as well as how this may have influenced the data analysis and selection of clusters. Both of these papers made valuable contributions towards understanding disclosure and non‐disclosure in a non‐Western supervisory dynamic. Throughout this review, the author took into account the potential limitations of these studies included in the analysis as per quality appraisals. While studies with lower quality ratings, such as Singh‐Pillay and Cartwright (2018) and Meydan (2020) did not endorse all concepts found in other studies, they made an equal contribution to the conceptual framework.

**TABLE 6:** CASP scoring for studies included.

Quality appraisal CASP question	Hess et al. (2008)	Sweeney and Creaner (2014)	Spence et al. (2014)	Cook et al. (2018)	Singh‐Pillay and Cartwright (2018)	Cook et al. (2019)	Foskett and Van Vliet (2020)	Meydan (2020)
1: Was there a clear statement of the aims of the research?	1	1	1	1	1	1	1	1
2: Is qualitative methodology appropriate?	1	1	1	1	0.5	1	1	1
3: Was the research design appropriate to address the aims of the research?	1	1	1	1	0.5	1	1	1
4: Was the recruitment strategy appropriate to the aims of the research?	1	1	1	1	1	1	1	1
5: Was the data collected in a way that address the research issue?	1	1	1	1	1	1	1	1
6: Has the relationship between researcher and participants been adequately considered?	1	1	1	1	1	1	1	0
7: Have ethical issues been taken into consideration?	1	1	1	1	0	1	1	0.5
8: Was the data analysis sufficiently rigorous?	1	1	1	1	1	1	1	0.5
9: Is there a clear statement of findings?	1	1	1	1	1	1	1	1
10a: Do the researchers discuss the contribution the study makes to existing knowledge or understanding?	Yes	Yes	Yes	Yes	Yes	Yes	Yes	Yes
10b: Do the researchers identify new areas where research is necessary?	Yes	Yes	Yes	Yes	Yes	Yes	Yes	Yes
10c: Do the researchers discuss whether or how the findings can be transferred to other populations or considered other ways the research may be used?	Yes	Yes	Yes	Yes	Yes	Yes	Yes	No
Total score (out of 9)	9	9	9	9	7	9	9	7

### 3.3 Results

**FIGURE 2:**
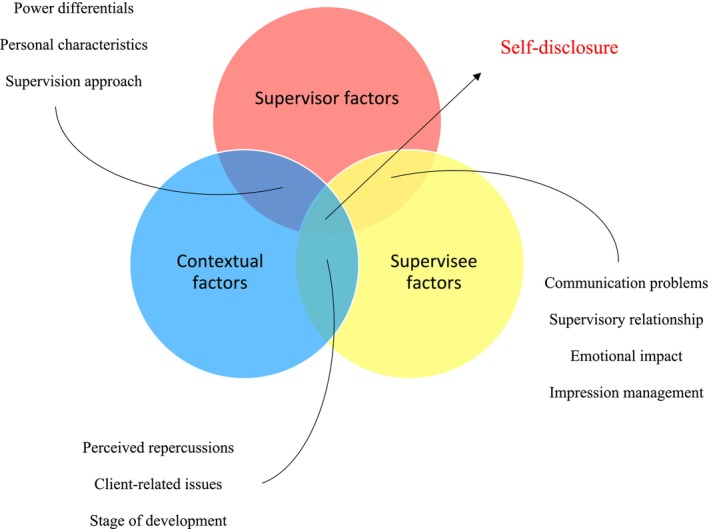
Conceptual framework of self‐disclosure.

Following Noblit and Hare (1988) seven‐stage methodology, the meta‐ethnography identified three meta‐themes (i.e., conceptual categories) encompassing 10 sub‐themes (i.e., third‐order constructs). To simplify the presentation of themes and their supporting quotes, sub‐themes have been categorised under their respective metathemes. However, there is an overlap, with each sub‐theme relating to two main themes due to the relational nature of supervision. Table 7 displays meta‐themes in primary colours (red, yellow, blue), with sub‐themes' colour coding indicating where they overlap (purple, orange, green), corresponding to the diagram presented in the overarching conceptual framework (Figure 2).

**TABLE 7:** Meta‐themes and sub‐themes.

Metathemes	Sub‐themes
Supervisor factors	Power differentials
	Personal characteristics
	Supervision approach
	Communication problems
	Supervisory relationship
Supervisee factors	Emotional impact
	Impression management
	Perceived repercussions
Contextual factors	Client‐related issues
	Stage of development

Table 8 shows the articles that contributed to each meta‐theme and sub‐theme. Colour coding was omitted in this table, to improve visibility for the reader.

**TABLE 8:** Meta‐themes and sub‐themes.

Study	Supervisor factors					Third‐order constructs			Contextual factors	
						Supervisee factors				
	Power differentials	Personal characteristics	Supervision approach	Communication problems	Supervisory relationship	Emotional impact	Impression management	Perceived repercussions	Client‐related issues	Stage of development
Hess et al. (2008)	✓	✓	✓	✓	✓	✓	✓	✓	✓	
Sweeney and Creaner (2014)	✓	✓	✓	✓	✓	✓	✓	✓	✓	✓
Spence et al. (2014)	✓	✓	✓	✓	✓	✓		✓	✓	✓
Cook et al. (2018)	✓	✓	✓	✓	✓	✓	✓	✓	✓	✓
Singh‐Pillay and Cartwright (2018)	✓	✓	✓		✓	✓	✓	✓	✓	✓
Cook et al. (2019)	✓	✓	✓	✓	✓	✓	✓	✓	✓	
Foskett and Van Vliet (2020)	✓	✓	✓	✓	✓	✓	✓	✓	✓	✓
Meydan (2020)	✓	✓	✓	✓	✓	✓	✓	✓	✓	✓

### 3.4 Supervisor Factors

This meta‐theme encompasses factors within the interpersonal dynamic between supervisors and supervisees, which supervisors must be mindful of during supervision. It emphasises the interplay between *power differentials*, supervisor's *personal characteristics*, *supervision approach*, *communication problems* and the *supervisory relationship*.

#### 3.4.1 Power Differentials

This sub‐theme emphasised supervisees' perception of their supervisor being ‘all‐knowing’ (Singh‐Pillay and Cartwright 2018, 87) and capable of passing judgement, leading to self‐doubt and non‐disclosure during clinical supervision:

I did not feel like it would be taken well, and that I am only an intern and should not correct her. (Cook et al. 2018, 122)

This is a course; after all, we will be graded. This also made me feel anxious. I was worried whether I would have to retake it and I did not disclose much. (Meydan 2020, 17)

Supervisees highlighted that supervisors evaluating their performance and having authority to hold them accountable negatively impacted self‐disclosure by triggering anxiety and fear of repercussions:

I know [the doctoral supervision of supervision course instructor] watches these videos and I feel like, whatever I say will go in there and they will tell [my course instructor] … sometimes I just feel uncomfortable with that … and I'm scared that it'll get back to [my course instructor]. (Cook et al. 2019, 212)

#### 3.4.2 Personal Characteristics

This sub‐theme shows the supervisees' perception of supervisors' personality traits. While supervisees acknowledge their own role in self‐disclosure during supervision, they want supervisors who show curiosity and empathy, providing room to discuss personal matters:

at the time there was a whole load of other things going on in my life, which I had told her about … and she kind of said, “If you're going to do this work, you have to get on with it and learn to put it aside.” So, I felt I couldn't even tell her that it was about being vulnerable. (Sweeney and Creaner 2014, 218)

Supervisees also hope for a less rigid supervisors and wish for them to be open to disclosing their own experiences, potentially serving as role models for self‐disclosure practices:

It was hard in the beginning, because of that feeling that I've got to disclose to you but I feel that you hold back and you are a very private person…. (Singh‐Pillay and Cartwright 2018, 88)

#### 3.4.3 Supervision Approach

This sub‐theme closely relates to power imbalances, influencing the character and efficacy of supervisory relationships. While some studies noted supervisees' preference for a ‘collaborative’ or ‘accommodating’ supervision style (e.g., Cook et al. 2019; Hess et al. 2008), two non‐Western studies identified an ‘authoritarian’ supervisory approach characterised by significant control and direction, limiting supervisees' autonomy and decision‐making:

When you get a supervisor who maybe sees themselves in a power relation, that's sometimes very difficult, because you don't want to disclose! “I'm the person who knows it all and I will tell you what to do, and if you don't do it my way then it's not the right way!” … so it really affects your professional development and your personal development. (Singh‐Pillay and Cartwright 2018, 87)

Some supervisees reported that supervisors who have taken an authoritative approach to supervision or those who have had negative attitudes towards self‐disclosure led to supervisees' non‐disclosure:

I more or less knew [my supervisor] from the past years. S/he did not seem to be able to change something. S/he was strict and reserved. Therefore, I felt unwilling to disclose. I thought I would not be understood. (Meydan 2020, 16)

#### 3.4.4 Communication Problems

Communication was integral to the supervisory experiences and was affected by several factors, such as unclear expectations and insufficient feedback. For instance, supervisees' uncertainty regarding the supervisory expectations may lead to non‐disclosure due to confusion about the supervision's purpose and requirements:

I'm pretty confused about what supervision is supposed to look like … if it was explained a little better in the beginning and expectations were set for supervision that would have been more helpful. (Cook et al. 2019, 211)

Supervisees have difficulties in expressing their needs for additional support from supervisors and raising dissatisfaction with the feedback received, perceiving it as more about how the supervisor would have handled the clinical work rather than providing constructive feedback:

I feel that I am not getting feedback about my counseling from my supervisor in the supervision meetings. Instead I am only getting suggestions of how the supervisor would have handled the client. (Cook et al. 2018, 121)

#### 3.4.5 Supervisory Relationship

This sub‐theme emphasises to the nature of supervisory relationship encompassing aspects, such as trust and openness between the supervisor and supervisee:

I felt that the relationship that I'd built up with my supervisor was a very good one, positive and very strong … I felt the relationship was based on trust and so I just felt I could go to him whatever. (Sweeney and Creaner 2014, 217)

Supervisees who felt safe to engage in self‐disclosure during clinical supervision described having ‘a very open supervision relationship’ and being ‘able to be relaxed in session’ (Foskett and Van Vliet 2020, 192). Those who struggle to trust their supervisor decided to withhold certain concerns that may have needed supervisory input:

… it was the openness which is key to creating an alliance that meant I could disclose appropriately. When you are not aware how things are happening, then it has a negative impact because you no longer trust your supervisor, and if you don't trust, it almost creates a cycle of non‐disclosure …. (Singh‐Pillay and Cartwright 2018, 88)

Further, supervisees' experiences showed that when there is alignment in professional values and theoretical orientation, it can cultivate a shared understanding and mutual goals between the supervisor and supervisee, thereby promoting self‐disclosure:

I think you have to have a similar attitude to what you do. I think it helps if you share a therapeutic model or there's at least an overlap in therapeutic model …. I would want people who had a similar way of looking at things … A similar sort of set of values I suppose. (Spence et al. 2014, 185)

### 3.5 Supervisee Factors

This meta‐theme includes the range of emotions, thoughts and perceptions supervisees experience within the supervisory context. It emphasises the sub‐themes of supervisees' *emotional impact* following self‐disclosure, *impression management* (i.e., their deliberate efforts made to shape or control supervisors' perception of them during clinical supervision) and *perceived repercussions*.

#### 3.5.1 Emotional Impact

Supervisees within the studies included highlighted the experience of feeling ‘uncomfortable’ or ‘unsafe’ (e.g., Sweeney and Creaner 2014, 215) after supervisors or colleagues learned something personal about them:

… afterwards it was always very awkward because they then knew something about me that I'd rather they didn't …. so I was then sort of walking around in my job feeling uncomfortable quite a lot of the time because they knew this and I think how they related to me from that day forward was different…. (Spence et al. 2014, 187)

Some supervisees felt that self‐disclosure led to supervisors criticising them ‘to the point of tears’ and expressed feeling unsafe due to contrasting clinical styles with the supervisor (Cook et al. 2018, 122). Also, there is a sense of ‘wrongfulness’ (Singh‐Pillay and Cartwright 2018, 89) and anticipation that supervisors make supervisees feel inferior, collectively illustrating the effect of supervisee's emotional experiences on disclosure:

I knew she would make me feel inferior. (Cook et al. 2018, 122)

#### 3.5.2 Impression Management

Supervisees admire their supervisors and see them as role models, aiming to reach similar levels of success. They may hesitate to take action or share information that could disrupt the supervisory relationship. They may fear that raising certain issues could alter the supervisor's ‘favourable opinion’ of them (Hess et al. 2008, 404). Thus, they prioritise preserving this positive perception and supervisory relationship over voicing their concerns:

I just think she is a really cool person and she is where I want to be at some point, so when it's somebody that I respect … I don't want to rock the boat in our relationship … I think she has a positive perception of me and I didn't want to change that. (Cook et al. 2019, 210)

Also, supervisees are hesitant to disclose certain information during supervision if they believe this could reflect negatively on their professional image. This fear indicates a concern about how their actions or decisions may be perceived by their supervisor and how it could potentially impact their career or supervisory relationships:

I would say I would be worried about and would have to think very hard about something, if I am concerned will put me in a bad light in terms of my profession, then I would be very nervous about disclosing that in supervision …. (Singh‐Pillay and Cartwright 2018, 86)

#### 3.5.3 Perceived Repercussions

Supervisees often weigh the advantages and disadvantages of disclosing difficulties before deciding whether to do so. They assess the potential negative consequences (‘costs’) against the potential positive outcomes (‘benefits’) to determine if the benefits outweigh the risks. Sometimes, they may decide that the risks of disclosure are too high unless immediate action is necessary:

I guess I weighed up the kind of costs of doing that … versus the benefits and I don't think there was any benefits that would make me feel the need to talk about it until a problem arose. (Spence et al. 2014, 186)

Further, supervisees often abstain from self‐disclosure including disagreeing with supervisor's feedback due to fear of potential negative reactions or misunderstanding as well as worrying that the information shared may be ‘used against’ them, leading to non‐disclosure or supervisee's ‘just making stuff up’ (Cook et al. 2019, 214):

It became very hard because I didn't feel particularly comfortable in it, so it became very hard to say … it became really hard because I was always fearing a negative reaction. (Sweeney and Creaner 2014, 217)

### 3.6 Contextual Factors

This meta‐theme refers to various contextual variables that could influence the supervisory process and outcomes. It includes two sub‐themes, including *client‐related issues* and the *stage of development* (of both supervisee and supervisor).

#### 3.6.1 Client‐Related Issues

Client‐related discussions are common in supervision. All the papers reviewed acknowledged supervisees' challenges related to client work, such as unprocessed client transference, and how these challenges affect self‐disclosure. Many supervisees experience ‘strong countertransference’ with clients (Cook et al. 2018, 121), making it hard to share with unreceptive supervisors:

… this countertransference stuff becomes difficult to share in supervision when you are dealing with a person who is not as open and you feel that you are going to be judged, and it goes beyond that to your own values and you have clashes sometimes …. (Singh‐Pillay and Cartwright 2018, 86)

When supervisors fail to create and nurture a safe and supportive environment for self‐disclosure, the supervisee risks not only distinguishing between their own emotions and those of their clients but also clients' safety:

Having a chronically suicidal client and … not assessing for SI in a session and feeling as if when assessed it was not done so well. (Cook et al. 2018, 121)

Disclosure in supervision becomes more comfortable when the supervisor prompts the supervisee to share their countertransference experiences or reactions to client issues:

It felt good to express that this is something I recognize [in me], and also I see in this other person [the client] …. It felt a bit gratifying to express that. (Foskett and Van Vliet 2020, 192)

When supervisor asked what you felt about an issue on the basis of our reactions to the client, disclosure became easier. (Meydan 2020, 14).

#### 3.6.2 Stage of Development

Supervisees experience growth and accomplishment through insights, reflections and feedback received during supervision sessions. Their stage of training and developmental level may reflect how they present themselves during supervision sessions (Meydan 2020). However, supervisors' agenda or practical variables such as the integration of both managerial and clinical supervision could influence their readiness to disclose:

… management supervision is pure and simple monitoring people's performance … all it is to check up that people can do the job, so it doesn't sit comfortably to have a person in that dual role because personally I don't want to disclose an awful lot about what I'm not doing very well. (Spence et al. 2014, 183)

Equally, supervisors who lack competence in addressing supervisee issues or fail to acknowledge their own limitations may encourage non‐disclosure among supervisees:

If your supervisor is not competent about the issues you are raising and cannot really tell you, “this isn't in my line,” and ends up mumbling around it, it's what you are taking out (as a professional) and it does reflect on your work because you will do exactly as taught. (Singh‐Pillay and Cartwright 2018, 88)

### 3.7 Conceptual Framework

The emerging conceptual framework provides new perspectives and highlights key factors that contribute to self‐disclosure in clinical supervision. The interplay between the supervisor factors, supervisee factors and contextual factors shapes supervisees' inclination for self‐disclosure (Figure 2):

At the core of the supervisory relationship are ‘supervisor factors’, which encompass the multifaceted role supervisors play in influencing the supervision process. This theme reflects how power differentials, personal characteristics, supervision approaches, communication effectiveness and the quality of the supervisory relationship impact supervisees' willingness to disclose. For instance, the perceived authority of supervisors often creates anxiety and fear of judgement among supervisees, hindering open communication and self‐disclosure. This anxiety is further exacerbated by a lack of empathy or curiosity from supervisors, emphasising the importance of a supportive and collaborative supervision style that fosters trust and openness. The supervisory relationship itself is pivotal; a positive, trusting relationship can significantly enhance a supervisee's comfort in sharing personal and professional challenges. Conversely, miscommunication or inadequate feedback can lead to misunderstandings and non‐disclosure, highlighting the necessity for clear, constructive communication within the supervisory context. Parallel to these supervisor‐related factors are ‘supervisee factors’, which delve into the supervisees' internal experiences and perceptions during supervision. Emotional responses following self‐disclosure, such as feelings of discomfort or fear of criticism, play a crucial role in determining whether supervisees continue to share openly. Additionally, concerns about maintaining a favourable impression and the potential repercussions of disclosure lead supervisees to carefully weigh the risks and benefits before deciding to disclose sensitive information. The interplay between these supervisor and supervisee factors is further influenced by ‘contextual factors’, such as client‐related issues and the developmental stages of both supervisors and supervisees. The challenges supervisees face in client work, including dealing with countertransference, can be particularly difficult to disclose if the supervisory environment does not feel safe or supportive. Moreover, the developmental stage of the supervisee influences their readiness to disclose, with more experienced supervisees potentially feeling more secure in sharing difficult issues. In essence, the effectiveness of supervision hinges on the alignment of these overlapping factors—supervisor, supervisee and contextual. When supervisors are mindful of the inherent power differentials, demonstrate empathy and cultivate an open, trusting relationship, they create an environment conducive to self‐disclosure. Similarly, when supervisees feel understood and supported, they are more likely to engage in the supervisory process fully, leading to self‐disclosure and more effective outcomes. Ultimately, a collaborative and accommodating supervision style, sensitive to the complexities of both the supervisee's emotional experiences and the broader contextual factors, is crucial for fostering an environment where self‐disclosure can thrive.

## 4 Discussion

This meta‐ethnography aimed to enhance prior qualitative systematic reviews (Chircop Coleiro et al. 2023; Falender et al. 2014) by providing a conceptual framework and new perspectives on the contributory factors that facilitate or prevent supervisee disclosure during clinical supervision. Self‐disclosure is an important aspect of effective supervision, enabling supervisors to fulfil their responsibilities of safeguarding the wellbeing of supervisees and clients, as well as fostering professional competence in clinical practice. However, research suggests that many supervisees continue to refrain from disclosing information during supervision due to various reasons, including fear of negative judgement, underestimating the significance of disclosure or perceiving the issue as too personal (Falender et al. 2014; Knox 2015; Kühne et al. 2019; Ladany et al. 1996; Mehr et al. 2010; Yourman and Farber 1996). This systematic review provides a comprehensive understanding of the factors influencing self‐disclosure within clinical supervision. The conceptual framework that emerged from the findings of this study outlines the nuanced interaction between the supervisor factors, supervisee factors and contextual factors, all of which contribute to supervisees tendency to engage in self‐disclosure. These findings align with the above‐mentioned reviews in the field, indicating that supervisors play a crucial role in encouraging and facilitating supervisee disclosure by responding sensitively and providing guidance, especially when disclosures involve evaluative components impacting supervisees' wellbeing and client care (Bradley and Becker 2021; Gibson et al. 2019; Kühne et al. 2019). This review highlights the complexity of the supervisory process and its impact on supervisee self‐disclosure as well as its implications for clinical supervision practice.

One of the overlapping themes identified in this study is the supervisor‐contextual factors, encompassing the power differentials, supervisor's personal characteristics (e.g., rigidity and lack of openness) and authoritarian supervisory approaches, all of which act as barriers to self‐disclosure due to fostering self‐doubt, feelings of apprehension and a sense of insecurity in supervisees. Acknowledging power differentials in clinical supervision is essential for fostering a safe learning environment for supervisees (Falender et al. 2014). Supervisees benefit from understanding the power dynamics inherent in the supervisory relationship as they become more aware of their own vulnerabilities and the influence of authority on their thoughts, feelings and behaviours (Borders et al. 2014). Current findings suggest that supervisees may feel intimidated or pressured to comply with their supervisor's directives, which can inhibit their ability to voice concerns or seek clarification. For instance, Singh‐Pillay and Cartwright (2018) found that supervisors who are perceived as authoritarian or rigid instil apprehension about self‐disclosure in supervisees. Conversely, a sincere and humorous supervisor may bring a sense of ease in supervisees, nurturing self‐disclosure (Meydan 2020). Wilson et al. (2016) support these findings, emphasising that supervisees fear supervisors' negative evaluation due to the power imbalance, which needs to be managed and discussed sensitively. Bernard and Goodyear (2014) emphasise that power dynamics can also be influenced by cultural factors such as age, gender, race and socioeconomic status. Although these factors were not deeply investigated within the studies included in this review, acknowledging them may allow supervisors to approach supervision with cultural sensitivity, recognising how societal power structures may impact the supervisory relationship between supervisors and supervisees.

The current findings regarding the overlapping supervisor–supervisee factors underscore the importance of adopting a collaborative and supportive supervisory dynamic, where supervisors acknowledge supervisees' internal experiences and welcome these with empathy and curiosity. Emotional reactions, such as feelings of discomfort and anticipation of negative repercussions, could significantly impact supervisees' willingness to disclose personal or sensitive information. Supervisors employing proactive approaches and diverse supervisory interventions (e.g., guided discovery, imagery and roleplay) may enhance supervisees' self‐awareness and cultivate a collaborative environment conducive to openly addressing and exploring concerns (Prasko et al. 2022; Shafranske and Falender 2008). These findings complement a recent descriptive and interpretative framework highlighting that supervisors need to be attuned to supervisees' emotional responses and create a supportive environment that encourages open dialogue of potential vulnerabilities (Chircop Coleiro et al. 2023). Concerns about impression management and the potential consequences of disclosure contribute to supervisees' reluctance to share certain issues with their supervisors. The current findings suggest that impression management also plays an important role in supervision, as it can influence both supervisors' and supervisees' perceptions, judgements and outcomes in supervisory interactions, affecting aspects such as trust and credibility (Singh‐Pillay and Cartwright 2018). Supervisees' desire to maintain a specific image or identity during supervision might trigger apprehensions in the supervisor regarding the lack of transparency in their practice. This, in turn, could lead supervisors to embrace a punitive or authoritarian supervisory approach, perpetuating a cycle of non‐disclosure among supervisees (An et al. 2020; Mohd Noor 2019).

The overlapping supervisor–supervisee factors encompass the interpersonal relationship between supervisors and supervisees within the context of supervision. Trust emerged as a critical component of this relationship dynamic, with supervisees highlighting the importance of feeling safe and supported within their supervisory relationships. This is consistent with the current literature, which acknowledges the necessity of supervisors creating a safe and supportive environment for supervisees (Chircop Coleiro et al. 2023; Wilson et al. 2016), increasing self‐disclosure occurrences (Foskett and Van Vliet 2020; Hess et al. 2008; Mehr et al. 2010; Singh‐Pillay and Cartwright 2018; Sweeney and Creaner 2014). Communication problems emerged as another significant interference factor to self‐disclosure, with supervisees expressing confusion about the expectations of supervision and dissatisfaction with the lack of feedback received from their supervisors. The findings of this study highlight that clear communication and feedback are essential for creating a conducive environment for self‐disclosure, as they enable supervisees to express their needs and manage expectations and concerns effectively. Falender et al. (2014) recommend that supervisors provide constructive feedback and establish clear expectations with supervisees to promote open communication barriers and engagement. Further, supervisees value supervisors who model self‐disclosure as a safe and standard practice, fostering openness and trust within the supervisory relationship. Multiple studies regarding supervisees' experiences with supervisor self‐disclosure confirm this, indicating that supervisor self‐disclosure strengthens the supervisory relationship and fosters supervisee self‐disclosure (Clevinger et al. 2019; Farber 2006; Knox et al. 2008; Knox et al. 2011).

The overlap between supervisee factors and contextual factors included perceived repercussions, stage of development and client‐related issues, further emphasising the complex nature of supervisee self‐disclosure. Supervision plays a crucial role in the professional development of supervisees, providing them with opportunities to grow, reflect and receive constructive feedback (Bradley and Becker 2021; Caras and Sandu 2014; Falender 2018; O'donovan et al. 2011). However, the current review shows that the way supervisees engage in supervision (including disclosure) may be driven by their stage of training and developmental level. Practical variables such as the integration of managerial and clinical supervision impact supervisees' inclination to engage in self‐disclosure. For example, the presence of a dual role supervisor who oversees both managerial and clinical aspects may hinder supervisees' willingness to disclose performance‐related vulnerabilities (Spence et al. 2014). On the other hand, supervisors' competence in addressing supervisees' concerns is also important, as those who fail to acknowledge their own limitations risk perpetuating a cycle of non‐disclosure among supervisees (Singh‐Pillay and Cartwright 2018). Finally, the study found that while discussions about clients and their presenting problems are fundamental in clinical supervision dialogues, supervisees often encounter difficulties in disclosing client‐related issues, particularly when facing countertransference. Supervisees expressed concerns about judgement and lack of receptivity from supervisors, hindering their willingness to share. Failure to create a safe and supportive environment for self‐disclosure not only affects supervisees' ability to distinguish between their emotions and those of their clients but also compromises client safety (Leary 2018). Nevertheless, when supervisors actively prompt supervisees to share their experiences of countertransference or reactions to client issues, disclosure may become more comfortable (Hess et al. 2008).

### 4.1 Limitations

This synthesis has achieved its aim, providing a conceptual framework concerning the contributory factors to supervisees' disclosure in clinical supervision. However, it is important to acknowledge that the included studies used various qualitative methods, presenting both challenges and opportunities. These differences in methodologies may have led to varied perspectives and information from participants due to discrepancies in interview procedures and analyses. For instance, a study employing the IPA approach (Smith et al. 2009) might elicit different responses compared to a grounded theory study due to their differing principles and amendment opportunities. The quality ratings of included studies were likely influenced by the chosen methodologies, with some lacking sufficient details about study procedures. Nevertheless, given the review's focus on interpreting second‐order constructs presented by the authors, the methodology employed for data collection was deemed appropriate. A notable methodological weakness was relying on a single reviewer throughout the synthesis, with only a few studies being assessed by a second, independent reviewer.

While Noblit and Hare's guidance (1998) does not specify the order in which to synthesise papers, the author organised the synthesis chronologically, aiming for ease throughout cross‐checking and aesthetics. However, the chronological order may have enriched current findings, particularly as researchers within the later studies reported more raw data (Cook et al. 2019; Foskett and Van Vliet 2020; Meydan 2020; Singh‐Pillay and Cartwright 2018). In hindsight, more chronological thinking at the earlier phases of meta‐ethnography could have provided clearer insights into evolving experiences and contributing factors for supervisees. With the exception of Singh‐Pillay and Cartwright (2018) and Meydan (2020), all included studies were conducted within Western cultures, raising concerns about potential cultural bias. The varying terminologies used to describe supervisees across the studies included suggest that non‐Western cultures may use terms unfamiliar to the author, possibly resulting in the exclusion of relevant studies. Additionally, some studies in this review lacked sufficient consideration of the researcher's role and its potential influence on data interpretation and theme identification, highlighting the need for increased reflexivity among researchers. Some studies lacked detail regarding the rationale behind specific analytical methods, which could be addressed to enhance methodological transparency.

Another limitation of the current research is that professional and ethical guidelines did not emerge in the results due to the limited consideration of these factors in the studies reviewed. However, it is important to acknowledge the crucial role these guidelines play in shaping the dynamics influencing supervisee self‐disclosure during supervision. Professional and ethical standards ensure that the supervision process adheres to established norms, promoting a safe, effective and ethical environment for both supervisors and supervisees. These standards include maintaining boundaries, addressing power imbalances and creating a non‐judgemental space where supervisees feel safe to disclose without fear of judgements or repercussions. By adhering to these guidelines, supervisors can mitigate the negative impact of power differentials and foster a more open and trusting supervisory relationship (Bernard and Goodyear 2014; Borders et al. 2014). Professional codes highlight the need for supervisors to demonstrate empathy, respect and openness, encouraging them to engage in self‐disclosure judiciously to model appropriate professional behaviour. Ethical recommendations also advocate for a collaborative, supportive supervision style, discouraging authoritative approaches to enhance supervisee autonomy and comfort in disclosing issues (Borders et al. 2014; Ladany et al. 2013). Furthermore, clear, transparent and honest communication,a cornerstone of ethical supervision,helps set clear expectations and provides constructive feedback, preventing misunderstandings and reducing supervisee anxiety or confusion, thus encouraging disclosure (Bernard and Goodyear 2014). In addition, these guidelines emphasise the importance of confidentiality and the appropriate handling of client information, creating a safe space for supervisees to discuss client‐related challenges without fear of breaching confidentiality. This, in turn, promotes better supervision outcomes (Bernard and Goodyear 2014). Tailoring supervision to the developmental needs of supervisees, providing appropriate support and being sensitive to supervisees' emotional wellbeing are also key elements that enhance the safety of disclosure (Ladany et al. 2013). Finally, ethical standards that promote honesty and transparency reduce the pressure on supervisees to manage impressions, allowing for more open and genuine communication (Bernard and Goodyear 2019).

### 4.2 Research Implications

Exploration of the experiences of supervisee disclosure and non‐disclosure in clinical supervision is limited, suggesting a suitable area for further investigation. Future research could explore the diversity of supervisees across cultures and delve into their individual experiences, gaining a more comprehensive understanding of non‐Western supervisory contexts. Also, future research could examine differences in experiences among supervisees' clinical versus managerial supervisions as well as those within NHS organisations versus third‐sector settings. Finally, future research could enhance the conceptual framework derived from this review by empirically testing its applicability in real‐world supervisory contexts. For example, conducting a quantitative study to explore how having different clinical and managerial supervisors, along with variations in their supervision training and styles, impacts self‐disclosure. Confirming the effectiveness of the framework could assist future guidelines in effectively promoting self‐disclosure in supervision.

### 4.3 Clinical Implications

The findings of this review indicate that supervisees' inclination for self‐disclosure depends on the synergy between the supervisory dynamic, contextual issues and their internal experiences, all presenting significant implications (Table 9).

**TABLE 9:** Clinical implications.

Type of implication	Details
Establishing trust and safety	Supervisors should foster a safe and supportive supervisory environment by building trust through empathetic listening and clear communication, and openness. Purposefully sharing their own experiences, challenges and insights could help to normalise and demonstrate self‐disclosure.
Setting clear expectations	Supervisors should establish clear practice guidelines regarding the appropriate use of self‐disclosure in supervision. This includes discussing when and how self‐disclosure may be used, all while maintaining professional boundaries and confidentiality.
Providing feedback	Supervisors should provide constructive feedback as needed, helping supervisees to develop a better understanding of the impact of self‐disclosure on the supervisory relationship and therapeutic processes.
Supervision training	Supervisors should remain updated on self‐disclosure best practices in supervision by attending ongoing professional development. Professional bodies should review current research and update policies on supervision and self‐disclosure practices accordingly.
Training programmes	Training programme should improve supervision training by including education on power dynamics within the supervisory relationship with trainees and offer workshops and support focused on developing skills for managing self‐disclosure in supervision.

## 5 Conclusion

Synthesising supervisees' perspectives on self‐disclosure in clinical supervision revealed that their tendency to disclose depends on the interplay between supervisor factors, supervisee factors and contextual factors. This systematic review findings hold significant implications for clinical supervision. Supervisee self‐disclosure depends on their supervisors cultivating trust, improving communication and supporting supervisee emotional wellbeing. Establishing a safe supervisory environment is vital for supervisee growth and development. Future research should continue to explore self‐disclosure dynamics in clinical supervision and identify effective interventions to enhance supervisee engagement and learning.

## Conflicts of Interest

The authors declare no conflicts of interest.

## Data Availability

Data supporting findings are available from the corresponding author upon sensible request.
